# An automated oxystat fermentation regime for microoxic cultivation of *Magnetospirillum gryphiswaldense*

**DOI:** 10.1186/s12934-020-01469-z

**Published:** 2020-11-10

**Authors:** Cornelius N. Riese, René Uebe, Sabine Rosenfeldt, Anna S. Schenk, Valérie Jérôme, Ruth Freitag, Dirk Schüler

**Affiliations:** 1 Department of Microbiology, University of Bayreuth, Universitätsstraße 30, 95447 Bayreuth, Germany; 2 Physical Chemistry I, University of Bayreuth, Universitätsstraße 30, Bayreuth, 95447 Germany; 3 Bavarian Polymer Institute (BPI), University of Bayreuth, Universitätsstraße 30, Bayreuth, 95447 Germany; 4 Physical Chemistry - Colloidal Systems, University of Bayreuth, Universitätsstraße 30, Bayreuth, 95447 Germany; 5 Chair for Process Biotechnology, University of Bayreuth, Universitätsstraße 30, Bayreuth, 95447 Germany

**Keywords:** Magnetosomes, *Magnetospirillum gryphiswaldense*, Oxystat fermentation, Magnetosome biomineralization

## Abstract

**Background:**

Magnetosomes produced by magnetotactic bacteria represent magnetic nanoparticles with unprecedented characteristics. However, their use in many biotechnological applications has so far been hampered by their challenging bioproduction at larger scales.

**Results:**

Here, we developed an oxystat batch fermentation regime for microoxic cultivation of *Magnetospirillum gryphiswaldense* in a 3 L bioreactor. An automated cascade regulation enabled highly reproducible growth over a wide range of precisely controlled oxygen concentrations (1–95% of air saturation). In addition, consumption of lactate as the carbon source and nitrate as alternative electron acceptor were monitored during cultivation. While nitrate became growth limiting during anaerobic growth, lactate was the growth limiting factor during microoxic cultivation. Analysis of microoxic magnetosome biomineralization by cellular iron content, magnetic response, transmission electron microscopy and small-angle X-ray scattering revealed magnetosomal magnetite crystals were highly uniform in size and shape.

**Conclusion:**

The fermentation regime established in this study facilitates stable oxygen control during culturing of *Magnetospirillum gryphiswaldense*. Further scale-up seems feasible by combining the stable oxygen control with feeding strategies employed in previous studies. Results of this study will facilitate the highly reproducible laboratory-scale bioproduction of magnetosomes for a diverse range of future applications in the fields of biotechnology and biomedicine.

## Background

Magnetosomes are membrane-enveloped magnetite (Fe_3_O_4_) or greigite (Fe_3_S_4_) crystals produced by magnetotactic bacteria for orientation along the Earth’s magnetic field [1]. Magnetosome biosynthesis is a complex, stepwise process involving the formation of a dedicated magnetosome vesicle, which serves as a nanoreactor for the subsequent uptake of iron and the biomineralization of magnetic crystals [2, 3].

In the widely studied alphaproteobacterium *Magnetospirillum gryphiswaldense* each step is highly regulated by a set of more than 30 genes leading to the formation of single crystalline magnetite particles with defined size, shape and magnetic properties, which are so far unmatched by magnetic nanoparticles produced by chemical synthesis [4–7]. Hence, magnetosomes are of great potential in the biomedical and biotechnological field, and isolated magnetosomes were already successfully applied for cancer treatment, such as magnetic hyperthermia [8–10], phototherapy [11] and radiosensitization [12], as contrast agent for magnetic imaging [13–16] and as a tool in immune assays [17]. Furthermore, the proteinaceous membrane of magnetosomes from *M. gryphiswaldense* and related organisms can be functionalized by genetic or chemical coupling of additional functional moieties, such as enzymes for immobilization of enzymatic cascades [18–21], fluorophores, antibodies for diagnostic purposes e.g. Immuno-PCR [20, 22–24], immunostimulatory ligands [25], or various inorganic and organic coating materials for enhancement of biocompatibility [26, 27].

Practical applications of magnetosomes have so far been limited by their poor availability, which is due to the lack of precise techniques for highly controlled large-scale production with defined process parameters. Mass cultivation of the available magnetobacterial strains is challenging due to their fastidious microaerophilic to anoxic lifestyle, and the dependence of magnetosome biosynthesis on suboxic conditions, which results in slow growth and low biomass as well as magnetosome yields in routine batch cultivation [1]. The most robust and widely used strain for magnetosome engineering and bioproduction is *M. gryphiswaldense*, which produces up to 60 cuboctahedral magnetite crystals with 20–50 nm in diameter [28, 29].

As for other magnetotactic bacteria, magnetite biomineralization in *M. gryphiswaldense* is strongly affected by growth conditions [30, 31]. While fastest growth occurs at moderately low oxygen partial pressures (pO_2_) of 0.25–2 mbar (equivalent to a dissolved oxygen (dO_2_) concentration of 0.1–1% of air saturation), magnetite biomineralization is increasingly stimulated by a decrease in oxygen concentration [30, 32]. Highest quantity and largest magnetite crystals are formed under denitrifying conditions in the entire absence of oxygen with nitrate (NO_3_^−^) as the only electron acceptor for respiration [30, 32–35]. However, anoxic flask cultivation to yields higher than about 0.3 OD_565_ is difficult due to the resulting toxic effects of denitrification intermediates such as nitrite (NO_2_^−^), which accumulate at NO_3_^−^ concentrations higher than 10 mM [33, 34]. This tradeoff can be alleviated by microaerobic cultivation, where oxygen and nitrate respiration overlap [32–35]. Nonetheless, the controlled microoxic conditions below 10% dO_2_, needed for optimal magnetosome production, are difficult to maintain and often require specific adaptations for the stable regulation, such as the programming of a reliable automated control cascade, and the bioreactor setup has to be characterized and optimized to enable homogenous mixing as well as precise and stable dO_2_ adjustment.

In the first systematic study, Heyen and Schüler [32] investigated growth and magnetosome biosynthesis of *M. gryphiswaldense*, *M. magnetotacticum* and *M. magneticum* in a 4 L bioreactor at various pO_2_ tensions from 0.25 mbar to 212 mbar (dO_2_ of 0.1% to 100% of air saturation) using an automated oxygen regulation with a specialized pO_2_ measurement and gas supply setup. They showed a clear correlation between pO_2_ and magnetosome biosynthesis in all three magnetospirilla and a maximum magnetosome yield of 7.9 mg L^−1^ was reached with *M. gryphiswaldense* at 0.25 mbar pO_2_ with a biomass yield of 0.4 g dry weight per liter (g_dw_ L^−1^) (OD_565_ 1,4) [32].

Sun et al. [36] applied a microoxic fed-batch oxystat strategy, where oxygen intake into the medium was regulated solely by agitation with a fixed airflow rate. Highest biomass and magnetosome yields of 2.17 g_dw_ L^−1^ (OD_565_ 7.24) and 41.7 mg L^−1^, respectively, were reached in a 42 L bioreactor, where stirrer speed was increased manually in increments of 40 rpm whenever the growth rate decreased markedly. Furthermore, dO_2_ was permanently kept below the dO_2_-probe’s sensitivity. In follow-up studies by Liu et al. [37] and Zhang et al. [38], the feeding strategy was further improved, resulting in biomass and magnetosome yields of OD_565_ 12.2 and 82.23 ± 5.36 mg L^−1^ up to 9.16 g_dw_ L^−1^ (OD_565_ 42) and 356.52 mg L^−1^, respectively. Again, in these studies, the stirrer speed was increased in pre-set time intervals independent of measured dO_2_. A different approach was applied by Fernandez-Castané et al. [39] in a 5 L bioreactor, where dO_2_ was controlled by manual adjustment of airflow between 0 and 0.1 standard liter per minute (SLPM) and agitation between 100 and 500 rpm, thereby keeping the dO_2_ permanently below the dO_2_-probe’s sensitivity (dO_2_ below 1% of air saturation). This resulted in biomass and magnetosome yields of OD_565_ 16.6 and 54.3 ± 0.4 mg L^−1^, respectively [39]. Moreover, cell morphology and viability were further investigated by flow cytometry, thereby showing viable cells throughout the fermentation process [40].

The most recent study conducted by Berny et al. [41] employed a manual regulation regime to cultivate *M. gryphiswaldense* in a minimal medium avoiding complex, non-defined constituents such as peptone and yeast extract. Maximal biomass and magnetosome yields were 2.4 g_dw_ L^−1^ (OD_565_ 8) and 10 mg L^−1^, respectively [41].

During an investigation of oxygen and iron impact on gene expression, an automated oxystat regime in a 1 L bioreactor based on dynamic gas blending of nitrogen and air was applied, whereas agitation and maximal gas flow remained constant at 120 rpm and 1 SLPM, respectively [42]. In this study, set dO_2_ values of 0.5% and 5% were kept stable over the fermentation, however this regime led to a steady decrease of oxygen concentration during growth at set dO_2_ values above 15% [42].

Despite the impressive improvements in *M. gryphiswaldense* fermentation, which resulted in ODs as high as 16–42 and magnetosome yields > 35 mg magnetite L^−1^ [38, 39], respectively, application has been hampered by the discontinuous dO_2_ control throughout cultivation. This in turn makes scale-up and transfer of protocols to other fermenter systems difficult, due to the highly specific, empirically determined parameters optimized for the particular type of bioreactor used in these studies. By contrast, an automated oxygen regulation regime would allow to overcome these limitations and thereby enhances reproducibility and handling through standardization of the process. Moreover, while it is well known that dissolved oxygen concentrations also greatly affect the size, shape and crystallinity of magnetite particles, previous studies mostly focused on the analysis and optimization of growth parameters rather than magnetosome characteristics. Typically, the *C*_mag_ (i.e. a light-scattering parameter for the semiquantative estimation of average magnetic alignment of cells [29]) and the intracellular iron content were used as proxies to quantify magnetosome bioproduction, even though these techniques do not provide precise information on the number, size and shape of magnetite particles. Finally, another important, but so far neglected aspect of process reproducibility is the preparation and treatment of precultures from stock to inoculation (i.e. the ‘seed-train’).

The aim of this study was the establishment and characterization of an oxystat fermentation regime for cultivation of *M. gryphiswaldense* employing an automatic cascade regulation for precise control of dO_2_, which was successfully applied for 95%, 10% and 1% dO_2_ values. Additionally, magnetosome biosynthesis was monitored throughout cultivation employing a combination of complementary analytical techniques including atomic absorption spectroscopy (AAS), quantitative transmission electron microscopy (TEM) and small-angle X-ray scattering (SAXS) [43] to study the composition and structure of the forming nanoparticles. To the best of our knowledge, the unique combination of complementary analytical techniques employed in this study provides the first truly quantitative assessment of magnetosome bioproduction at well-defined oxygen concentrations, thus enhancing the understanding of oxygen impact in magnetite biomineralization.

## Results

### Establishment of an automated oxystat fermentation regime

**Fig. 1:**
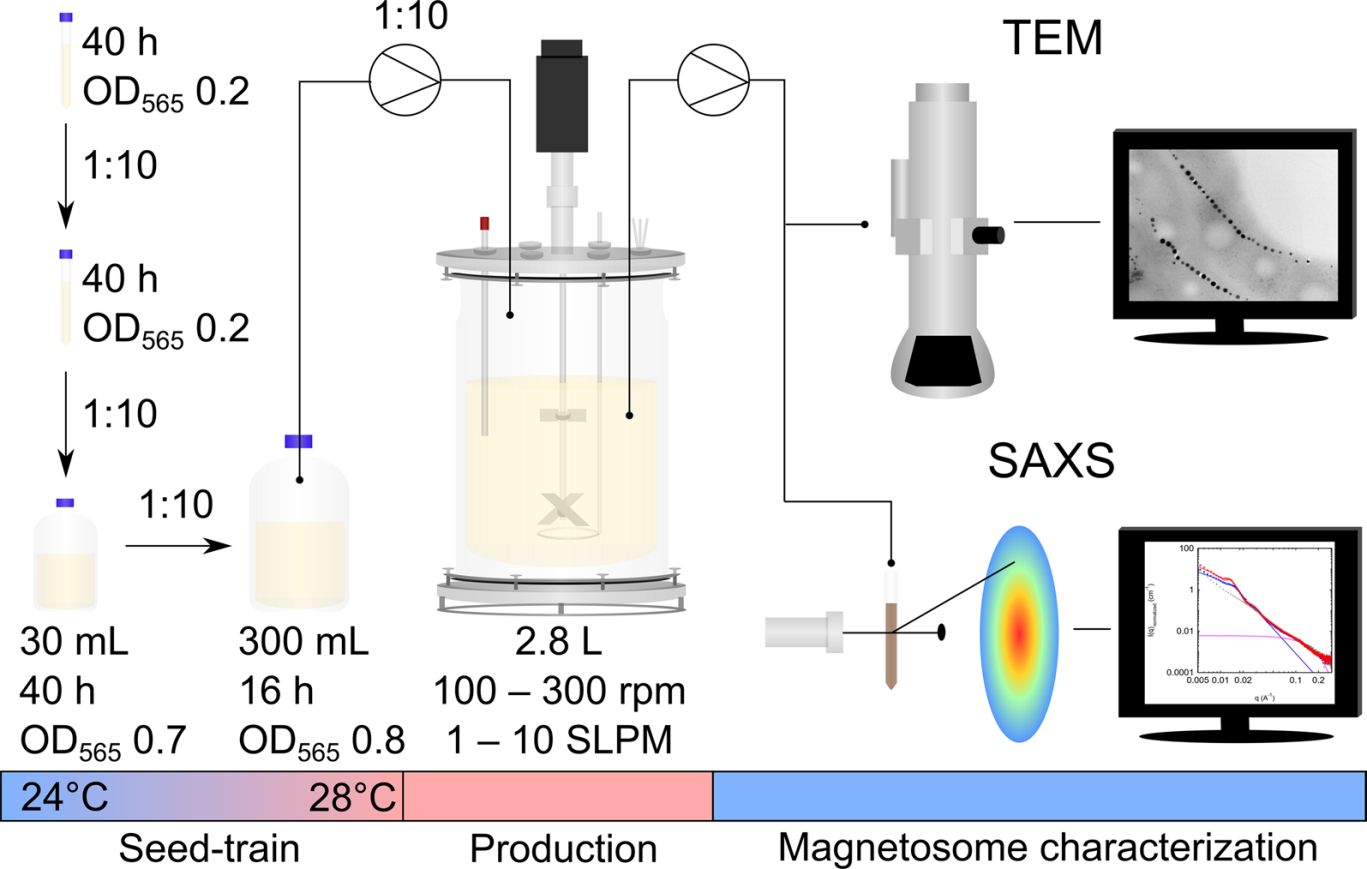
Overview over the oxystat fermentation approach including seed-train, production with different stirrer speeds (rounds per minute = rpm) as well as airflow rates (standard liter per minute = SLPM) and magnetosome characterization by transmission electron microscopy (TEM) and small-angle X-ray scattering (SAXS). See text for more details

The seed-train used in this study encompassed two passages for initial subcultures incubated in 10 mL at room temperature (24 °C) for 40 h. Further subcultivation was performed by step-wise scale-up in screw-capped bottles of different sizes to reach the final inoculum of 300 mL at an OD_565_ of 0.8 (equivalent to about 1.8 × 10^8^ cells mL^−1^) after 16 h with magnetic (*C*_mag_ = 1.20), highly viable cells (as judged by their motility), encompassing ~ 15 generations from stock to final inoculum. This seed-train (as summarized in Fig. 1) was used in all subsequent fermentation experiments.

To ensure optimal and reproducible oxygen input and dispersion, we first characterized and optimized the setup of the 3 L bioreactor. Mixing and oxygen transfer could be greatly improved by combining a radial (Rushton) and axial mixing (pitched blade) impeller type [44].

**Fig. 2:**
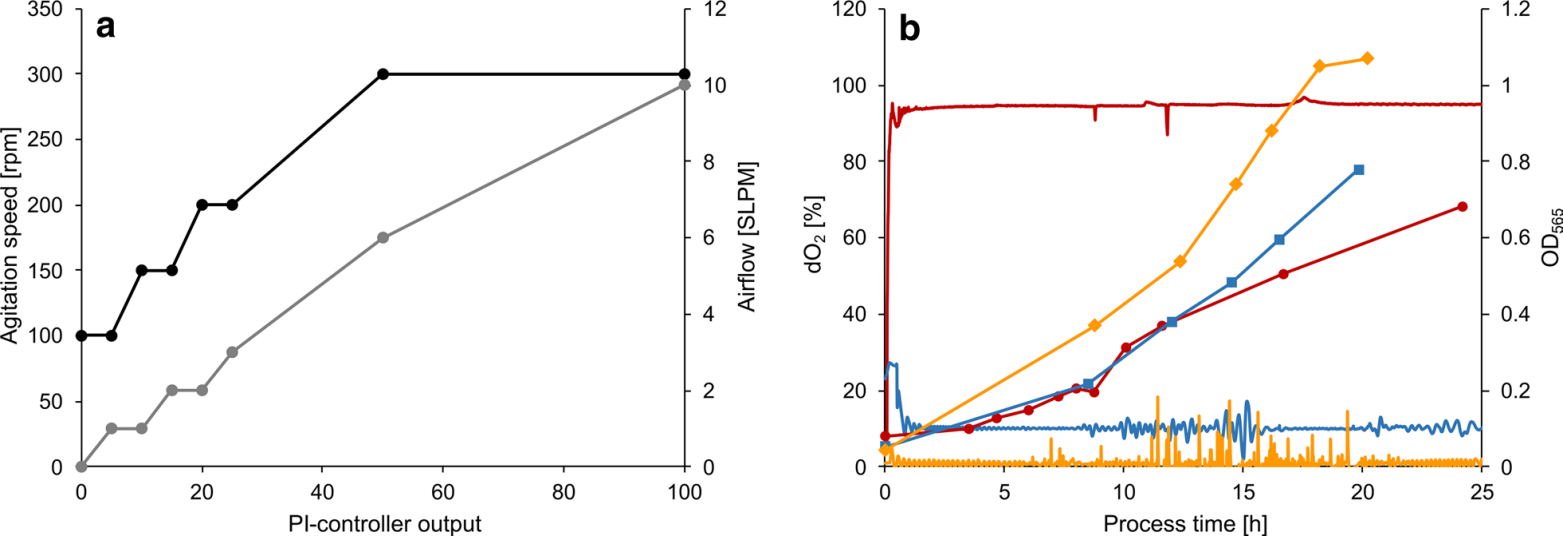
**a** Programmed cascade for automated dO_2_ control by dynamic adjustment of agitation (black) and aeration (grey) with compressed air (21% dO_2_). **b** Representative fermentations at 95% (red circles), 10% (blue squares) and 1% (orange diamonds) dO_2_. OD_565_ is indicated by filled symbols. dO_2_ is indicated by solid lines

Next, we aimed to establish a controlled oxystat fermentation regime for a wide range of oxygen concentrations. To ensure constant dO_2_ throughout growth, we used a *Proportional Integral* (PI) controller mediated cascade for automated regulation by combining control of airflow and stirrer speed. The cascade was programmed to match the requirements that both stirrer speed and airflow have to precisely maintained at low levels in the beginning, but need to be sufficiently high to sustain increasing cell densities throughout cultivation. Accordingly, stirring as well as airflow were increased in a stepwise manner independently from each other, exclusively regulated by the PI-controller output (Fig. 2a). To test the precision of the optimized dO_2_ regulation, fermentations were performed at oxic (dO_2_ 95%) and microoxic (dO_2_ 10% and 1%) conditions.

With a set value of 95% (200 mbar), dO_2_ was maintained precisely over the entire cultivation, with only minor fluctuations (median of 94.9%) (Fig. 2b). The final OD_565_ of 0.76 was reached after 30 h, but as expected only a weak *C*_mag_ of 0.1 was detectable, due to known inhibition of magnetite biomineralization by dO_2_ above 5% (10 mbar) [32].

At 10% (20 mbar) set dO_2_ value, monitored oxygen was consistently stable with a median of 10% dO_2_. Larger fluctuations at 10% dO_2_ were observed during main growth phase between 10–16 h of incubation, where the control cascade has to cope with increasing oxygen requirements of the culture (Fig. 2b). Although growth was greatly enhanced (maximal OD_565_ of 1.2 after 35 h) compared to oxic conditions (95% dO_2_), magnetosome biomineralization was still inhibited as indicated by a low *C*_mag_ value of 0.1 at the end of process, similar as previously described [32].

At 1% (2 mbar) set dO_2_ value, the median of measured dO_2_ was 0.9%. Here, most of the sporadically occurring fluctuations in dO_2_ were also observed during main growth phase between 11–20 h of incubation. These fluctuations result most likely by a combination of both probe sensitivity, visible by higher background fluctuations at 1% dO_2_ in comparison to higher set dO_2_ values and cascade reactivity, indicated by a higher fluctuation frequency during the main growth phase. Despite of the sporadic dO_2_ fluctuations (Fig. 2b), growth and magnetosome formation (*C*_mag_) were consistent in duplicate experiments with maximal OD_565_ of 1.1 after 18 h and highest *C*_mag_ of 0.7 (Additional file 1: Figure S2). Since at dO_2_ concentrations below 1%, the frequency of these sporadic fluctuations would increase, thereby disrupting stable oxygen control, 1% dO_2_ was investigated as lowest oxygen condition in the following experiments.

### Effect of the oxygen concentration on growth, substrate consumption and biomineralization

In order to estimate the effect of different dO_2_ tensions on growth and magnetosome biomineralization, we performed batch fermentations in biological triplicates and with independent seed-trains at oxic (95% dO_2_) and microoxic (1% dO_2_) conditions. For comparison, anoxic (0% dO_2_) fermentations were performed, which were expected to sustain optimal magnetosome biosynthesis. For anoxic (denitrifying) growth, the concentration of nitrate as electron acceptor was increased to 10 mM to enable higher cell yields, while 4 mM nitrate was supplemented at microoxic and oxic growth conditions.

**Fig. 3:**
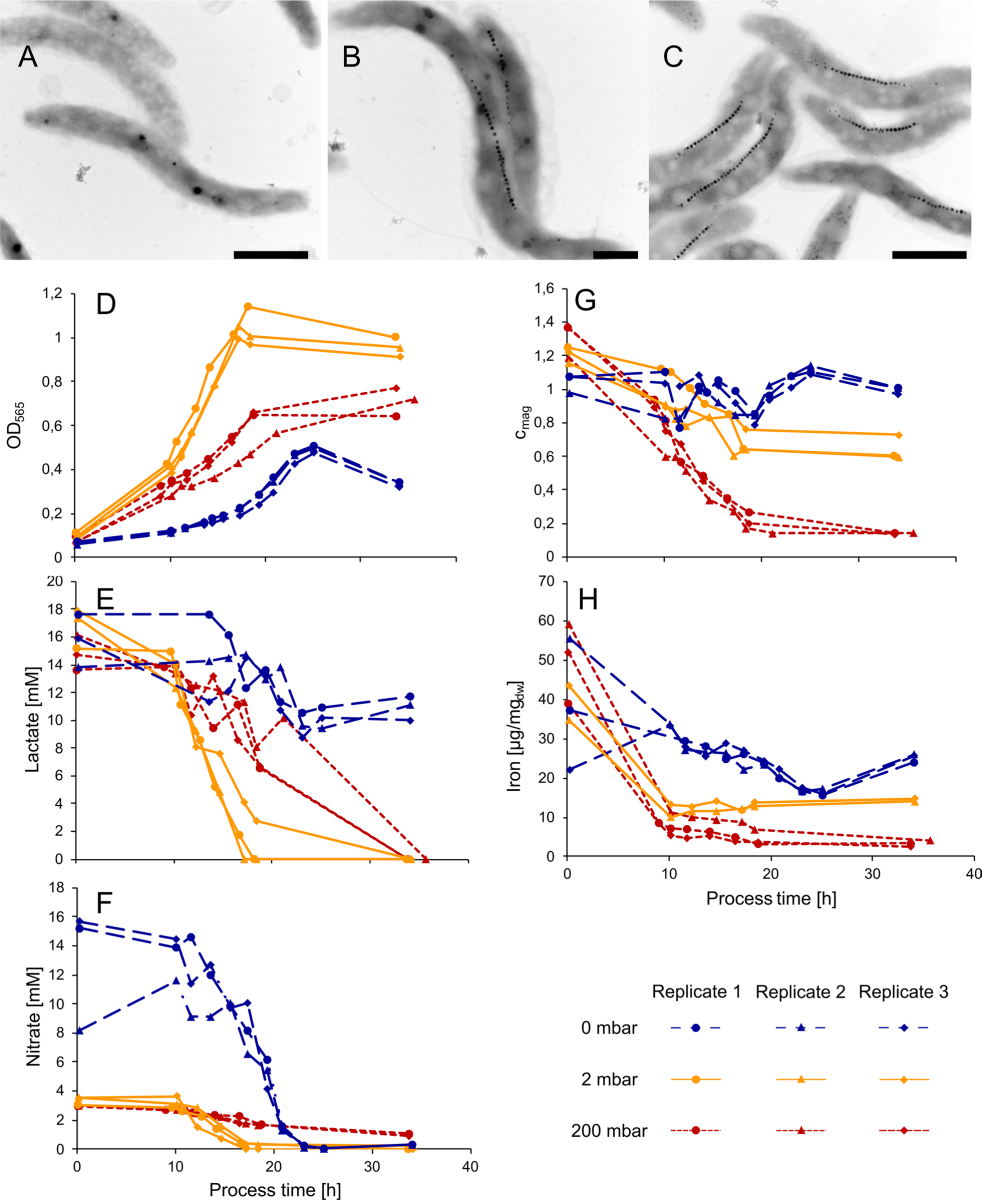
TEM micrographs of cells grown under **a** oxic (95% dO_2_, scalebar 1 µm), **b** microoxic (1% dO_2_, scalebar, 500 nm) and **b** anoxic (0% dO_2_, scalebar 1 µm) conditions. Growth and substrate consumption triplicates at dO_2_ tensions of 95% (red), 1% (orange) and 0% (blue). **d** Growth by OD_565_. **e** Lactate concentration in mM. **f** Nitrate concentration in mM. **g** Magnetic response *C*_mag_. **h** Iron content of the cell pellet in µg mg_dw_^−1^

Under all tested conditions, growth was highly consistent among replicates as shown in Fig. 3d. As observed before, growth was lowest at 95% dO_2_ with a growth rate of 0.07 ± 0.009 h^−1^ (doubling time of 9.61 ± 1.3 h). The maximal OD_565_ under this condition of 0.71 ± 0.05 was reached after 35 h. Maximal growth among all tested conditions was observed at 1% dO_2_ with a growth rate of 0.15 ± 0.007 h^−1^ (doubling time of 4.76 ± 0.23 h) and a maximal OD_565_ of 1.06 ± 0.06 after ca. 18 h. In the absence of O_2_ (0% dO_2_), the growth rate was reduced to 0.13 ± 0.005 h^−1^ (doubling time of 5.24 ± 0.21 h) with a maximal OD_565_ of 0.49 ± 0.01 after 25 h. In all three cases, cells were highly motile (i.e. viable) and showed apparently identical sizes and helical cell shapes at the end of growth (average cell length of 3.4 ± 0.9 µm) (Fig. 3a–c).

Consistent with the observed growth rates, lactate as the main carbon source and nitrate as the main nitrogen source as well as the main electron acceptor under anoxic and oxygen-limited conditions were depleted from the medium with different rates during the main growth phase (Fig. 3e, f). Both oxic (95% dO_2_) and anoxic (0% dO_2_) cultures showed low lactate consumption rates with 2.67 ± 1.17 and 3.12 ± 1.17 mM h^−1^ OD_565_^−1^, respectively. The highest lactate consumption was observed under microoxic (1% dO_2_) conditions with 4.76 ± 0.23 mM h^−1^ OD_565_^−1^, whereas biomass yields in mg dry-weight per mmol substrate (Y_x/s_) did not significantly differ between oxygen conditions (Table 1).

**Table 1:** Growth and substrate consumption rates of cells during main growth phase at 95% (12–18 h), 1% (10–17 h) and 0% (17–23 h) dO_2_

	95% dO_2_	1% dO_2_	0% dO_2_
Growth rate µ [h^−1^]	0.07 ± 0.009	0.15 ± 0.007	0.13 ± 0.005
Doubling time [h]	9.61 ± 1.3	4.76 ± 0.23	5.24 ± 0.21
Maximal OD_565_	0.71 ± 0.05	1.06 ± 0.06	0.49 ± 0.01
Lactate consumption rate [mM h^−1^ OD_565_^−1^]	2.67 ± 1.17	4.63 ± 1.02	3.12 ± 1.17
Y_x/s_ [mg_dw_ mmol_lactate_^−1^]	4.59 ± 0.88	4.08 ± 0.43	5.40 ± 1.04
Nitrate consumption rate [mM h^−1^ OD_565_^−1^]	0.71 ± 0.28	1.09 ± 0.15	6.13 ± 0.83
Y_x/s_ [mg_dw_ mmol_nitrate_^−1^]	26.5 ± 4.6	21.1 ± 4.2	2.5 ± 0.9
Cellular iron content [mg g_dw_^−1^]	3.3 ± 0.7	14.4 ± 0.4	25.3 ± 0.9

Rates were measured in biological triplicates. Iron content of the biomass was measured after the end of cultivation at 35 h. Biomass productivity per mol substrate for lactate and nitrate (Y_x/s_) was calculated for the end of growth, where OD_565_ reached the maximum

The lowest NO_3_^−^ consumption was observed during aerobic growth (0.71 ± 0.28 mM h^−1^ OD_565_^−1^), because here nitrate serves only as nitrogen source, due to repression of respiratory nitrate reduction by O_2_ as electron acceptor (Fig. 3f) [33, 34].

At low (1%) dO_2_ tensions, nitrate as well as oxygen were both simultaneously used as electron acceptors for respiration [34, 35], which is consistent with an increased NO_3_^−^ consumption of 1.09 ± 0.15 mM h^−1^ OD_565_^−1^ (Table 1).

Highest nitrate consumption was observed during anaerobic growth. After 25 h, nitrate initially present at 10 mM was completely consumed (6.13 ± 0.83 mM h^−1^ OD_565_^−1^), resulting in limited growth under these conditions as indicated by the early onset of the stationary phase marked by the slight decrease of OD_565_ as typically observed for *M. gryphiswaldense* (Fig. 3d, f). Although a clear correlation between growth and nitrate concentration was observed, the use of a higher initial amount of nitrate is prohibited by the accumulation of toxic intermediates during denitrification [33, 34]. Alternatively, addition of nitrate upon depletion could theoretically prolong the main growth phase, since substrates such as lactate and iron are still sufficiently present in the fermentation medium.

**Fig. 4:**
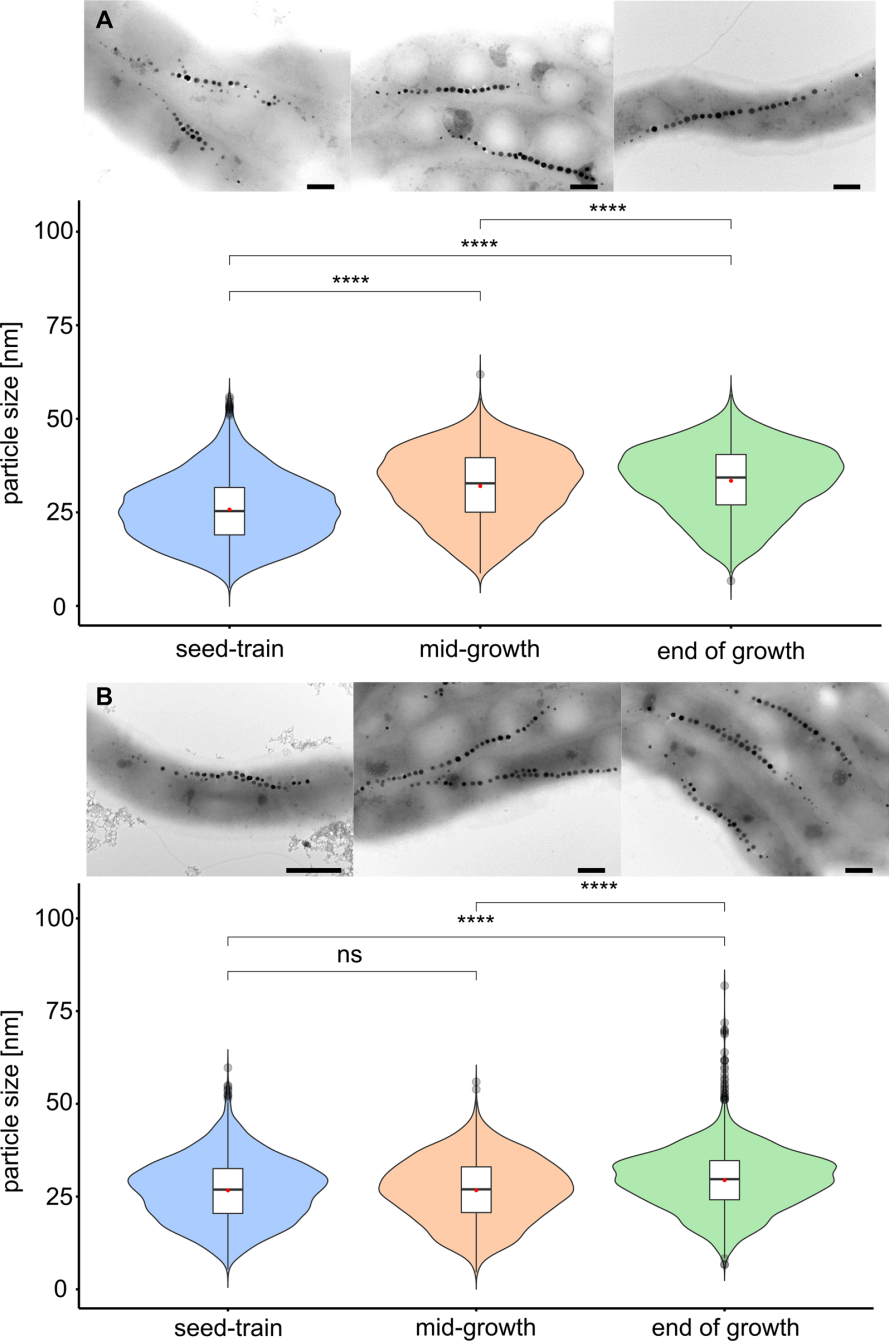
**a** Transmission electron micrographs of representative cells (scale bar 200 nm) and magnetosome particle sizes under anoxic (0% dO_2_) conditions at different timepoints of the process. Seed-train = 0 h (*n *= 2925), mid-growth = 15 h (*n *= 3069) and end of growth = 20 h (*n *= 3058). **b** Transmission electron micrographs (scale bar 1 µm (left micrograph) and 200 nm) and magnetosome particle sizes under microoxic (1% dO_2_) conditions at different timepoints of the process. Seed-train = 0 h (*n *= 3421), mid-growth = 14 h (*n *= 2267) and end of growth = 18 h (*n *= 3180). In box plots, the box indicates the interquartile range, the bar indicates the median, and the red dot represents the mean. Grey dots represent data points above or below the 95th and 5th percentile. The violin plots show the magnetosome particle size distribution of measured particle sizes. For statistical comparison of particle sizes, Wilcoxon rank sum test was performed (****, p < 0.0001; ns, not significant)

For evaluation of the oxygen impact on magnetite biomineralization, we characterized magnetosome quality at 95%, 1% and 0% dO_2_ by the *at line* techniques *C*_mag_ and AAS (Fig. 3g, h) as well as the quantitative techniques TEM and SAXS (Additional file 2: Figure S3). Different oxygen conditions had a clear effect on biomineralization: A *C*_mag_ value of 0.99 ± 0.01 was measured under anoxic conditions, indicating optimal magnetosome production of the culture, which was also confirmed by TEM (Fig. 4a, TEM). Here, largest magnetosome particles were observed with average diameters of 33.8 ± 9.4 nm at the end of the process after 34 h. At the start of growth, magnetosome particle sizes originating from the microoxic inoculum were at 25.8 ± 8.9 nm with a wide distribution including both smaller and larger particles (Fig. 4a, seed-train). Already after 15 h in mid-growth phase, particle sizes steadily increased to 32.0 ± 9.5 nm with fewer smaller particles (Fig. 4a, mid-growth). At the end of growth after 20 h, a further increase to 33.5 ± 9.1 nm was observed (Table 2), whereas longer incubation did not lead to a significant increase in magnetosome size, thus marking the maximum of magnetosome particle sizes in the batch fermentation experiment (Fig. 4a, end of growth). To further estimate O_2_ effects on biomineralization during cell elongation, the determined average magnetosome number per cell was normalized to cell length. Throughout cultivation numbers of magnetosomes remained constant at around 25 with approximately 8 magnetosomes (MS) per µm cell length (= MS µm^−1^). Only in mid-growth phase, a slight increase of around 0.7 MS µm^−1^ was observed, most likely caused by smaller cells during faster division in this growth phase (Table 2). Additionally, particle morphology was much more homogeneous at the end of the process in comparison to the start (Fig. 4a, TEMs). Overall magnetite crystals appeared more evenly shaped under anoxic conditions. Consistently, throughout growth also the intracellular iron content increased steadily, reaching up to 25.3 ± 0.9 mg g_dw_^−1^ (Fig. 3h).

**Table 2:** Characteristics of magnetosomes produced under anoxic and microoxic conditions

Process time		MS diameter [nm]	Cell length [µm]	MS/Cell	MS/cell length [µm^−1^]
Anoxic					
0 h		25.8 ± 8.9	3.0 ± 0.7	24.4 ± 2.3	8.1 ± 0.5
15 h		32.0 ± 9.5	2.9 ± 0.6	25.6 ± 0.6	8.8 ± 0.8
20 h		33.5 ± 9.1	3.0 ± 0.8	25.5 ± 2.1	8.4 ± 0.2
34 h		33.8 ± 9.4	3.1 ± 0.7	25.3 ± 0.6	8.1 ± 0.2
Microoxic					
0 h		26.7 ± 8.5	3.3 ± 0.8	29.2 ± 2.7	8.8 ± 0.1
14 h		26.7 ± 8.4	3.1 ± 0.8	27.9 ± 0.9	8.9 ± 1.1
18 h		29.3 ± 7.9	3.4 ± 0.9	28.4 ± 2.8	7.7 ± 0.6

Magnetosome diameter was measured with approximately 1000 particles per triplicate. Cell length was determined for 100 cells

At 1% dO_2_ a medium *C*_mag_ of 0.74 ± 0.007 was measured. Accordingly, microoxic conditions led to intermediate-sized magnetosome particles of 29.3 ± 7.9 nm in comparison to anoxic and oxic conditions (Table 2). Here, particle sizes increased throughout the fermentation process. Seed-train cultures under uncontrolled dO_2_ conditions showed particle sizes of 26.7 ± 8.5 nm (Fig. 4b, seed-train). In mid-growth phase after 14 h of incubation, average particle diameter was 26.7 ± 8.4 nm (Fig. 4b, mid-growth), but reached a maximum of 29.3 ± 7.9 nm after 18 h incubation. Due to the fact that no further increase in magnetosome size was observed under anoxic conditions as optimal magnetosome forming conditions between mid-growth and stationary phase, final magnetosome sizes were investigated at the end of the main growth phase. Cells contained ca. 28 magnetosome particles per cell with ca. 8 MS µm^−1^. Furthermore, particle and chain morphology became more regular throughout the process (Fig. 4b, TEMs). The intracellular iron content steadily increased up to 14.4 ± 0.4 mg g_dw_^−1^ (Fig. 3h).

Oxic (95% dO_2_) conditions entirely abolished magnetosome bioproduction, as indicated by a steady decrease of *C*_mag_ down to nearly 0 (Table 3). Additionally, in TEM analyses only 10% of the cells showed up to three magnetosomes per cell likely originating from the microoxic inoculum. Further a steady decrease of iron content in the biomass was detected to 3.3 ± 0.7 mg g_dw_^−1^ (Fig. 3h).

**Table 3:** *C*_mag_ and magnetite particle sizes measured by quantitative TEM and SAXS under the three tested dO_2_ conditions at the end of cultivation after 34 h (95% dO_2_),18 h (1% dO_2_) and 34 h (0% dO_2_)

	*C*_mag_	TEM		SAXS [nm]
		Median [nm]	Average diameter [nm]	
Oxic (95% dO_2_)	0.13 ± 0.005	–	–	–
Microoxic (1% dO_2_)	0.74 ± 0.07	29.7	29.3 ± 7.9	30.6 ± 7
Anoxic (0% dO_2_)	0.99 ± 0.01	35.0	33.8 ± 9.4	35.6 ± 7

To further verify average magnetosome sizes determined by TEM image analysis, we applied SAXS as a volume-sensitive non-destructive bulk technique for the quantitative assessment of nanostructural parameters. Most importantly, SAXS analysis enables us to extract the radii of both, the magnetosome core (R_core_) and the surrounding magnetosome membrane (R_membrane_), while avoiding possible artifacts caused by sample preparation.

Average magnetosome radii (R, R = R_core_ + R_membrane_) analyzed by SAXS were derived from the first form factor minimum at q ≈ 0.03 Å^−1^ resulting in overall magnetosome diameters of 2R = 39 ± 7 nm for 0% dO_2_ and 2R = 34 ± 7 nm for 1% dO_2_ (both Gaussian size distribution, Additional file 2: Figure S3 A solid blue and yellow line). The contribution of surrounding vesicles to the magnetosome radius R was estimated by modeling the Guinier law representative of small spherical objects to the SAXS profile of aerobically cultivated samples, which do contain little or no magnetite within the empty vesicle. In this model, the proteinaceous vesicle membrane is regarded as an envelope composed of spherical protein structures with radii smaller than the magnetite core (Additional file 2: Figure S3 B, solid green line). Since the sharp phase boundary in the excess electron density distribution between magnetite crystal and its surrounding membrane blurs in polydisperse multi-particle systems, the membrane thickness of R_membrane_ = 1.7 nm estimated from the radius of gyration (R_g_ = 1.3 nm), has to be subtracted prior to comparison with TEM data, which are predominantly sensitive to the high contrast magnetite core. Taking the thickness of the biomembrane into account, the dimensions extracted from SAXS measurements are in very good agreement with the TEM results (Table 3).

## Discussion

Since optimum growth and magnetosome biosynthesis only occur within a narrow range of oxygen concentration, reproducibility of both greatly suffers from unstable O_2_ control. In this study we established a well characterized, automated oxystat fermentation regime, which enables both reproducible cultivation of *M. gryphiswaldense* over several oxygen conditions (0%, 1% and 95% dO_2_) as well as highly uniform magnetosome production. The regime was designed to solve three major limitations: First, a standardized microoxic seed-train procedure was developed to enhance process stability by inoculation of the bioreactor with highly viable cells resulting in highly reproducible growth behavior among biological triplicates (Fig. 3a). Second, a cultivation process with an automated oxygen control cascade was designed and successfully applied for stable regulation of oxic and microoxic dO_2_ concentrations. Even at 1% as lowest dO_2_ condition tested, cultures showed highly consistent growth behavior (Additional file 1: Figure S2). Third, a comprehensive analytical workflow for magnetosome particle characterization combining a variety of different techniques was established for evaluation of magnetosome quality. Additionally, magnetosome production was assessed by theoretically calculated productivities of pure magnetite based on intracellular iron measurements.

Our automated cultivation regime thus enabled higher reproducibility and more precise regulation of cultivation conditions in comparison to manually controlled fermentation regimes [36–39, 41]. This was achieved by a dynamic response to changing oxygen requirements of the culture using an up-to-date fermentation system with standard oxygen electrodes and thermal mass flow controllers for gas inlet instead of specialized custom bioreactor modifications [32].

As already observed in previous studies [30, 32] differences in growth and magnetosome biosynthesis between O_2_ conditions were confirmed. Maximal growth rates during the main growth phase were reached at 1% dO_2_. Growth became limited by the depletion of lactate, whereas the depletion of nitrate could be compensated by oxygen as electron acceptor and other nitrogen sources.

With increasing oxygen concentration, growth became impaired by oxidative stress, as indicated by a decrease in growth rate at 95% dO_2_. Again, growth was inhibited by lactate depletion, whereas nitrate consumption was much lower because of repression of denitrification by high oxygen access regulated by a homologue of the oxygen-sensing transcription factor Fnr called MgFnr [34]. In *M. gryphiswaldense* nitrate reduction is the only pathway for energy production instead of oxygen respiration in anoxia resulting in rapid nitrate depletion [33]. Accordingly, anoxic growth was limited by nitrate depletion in the growth medium. Additionally, weakest growth was observed under anoxic conditions, caused by consumption of nitrate as the sole electron acceptor [33]. Since the results show rapid nitrate depletion and increasing the initial nitrate concentration is limited due to the accumulation of toxic intermediates in batch processes, a way to increase biomass yield would be a dynamic nitrate feeding strategy in future studies. While ammonium seems to be the preferred nitrogen source for several bacteria like *Escherichia coli* [45, 46], nitrate enabled highest growth and magnetite yields in magnetospirilla under microoxic conditions [33, 47].

The main scope of our study was the automation of the process and characterization of magnetosome parameters, rather optimization of high cell yields. Indeed, despite of the high reproducibility of our method, yields of biomass were still substantially lower (1 OD_565_) compared to previous fed-batch processes (14–42 OD_565_) [38, 39]. However, by employing feeding strategies based on the substrate consumption rates determined in our study, and an improved understanding of the metabolic mechanisms affecting cell growth and magnetosome formation, e.g. by metabolomic studies of *M. gryphiswaldense* [48], also biomass production can likely be substantially increased in future efforts using our oxystat regime.

Oxically grown cells (95% dO_2_) were essentially devoid of magnetosomes and displayed a *C*_mag_ of nearly 0 and a cellular iron content of 3.3 mg g_dw_^−1^. Decreasing the dO_2_ led to initiation of magnetosome biomineralization with an iron content of 14.4 mg_iron_ g_dw_^−1^ at 1% dO_2_ and 25.3 mg_iron_ g_dw_^−1^ at 0% dO_2_ marking maximal magnetosome production. The size of magnetite particles increased during anoxic and microoxic fermenter growth, as well as the uniformity of both size and morphology, whereas uncontrolled oxygen conditions, such as in shaking flasks, led to a much wider distribution in magnetite particle diameter (Fig. 4).

**Fig. 5:**
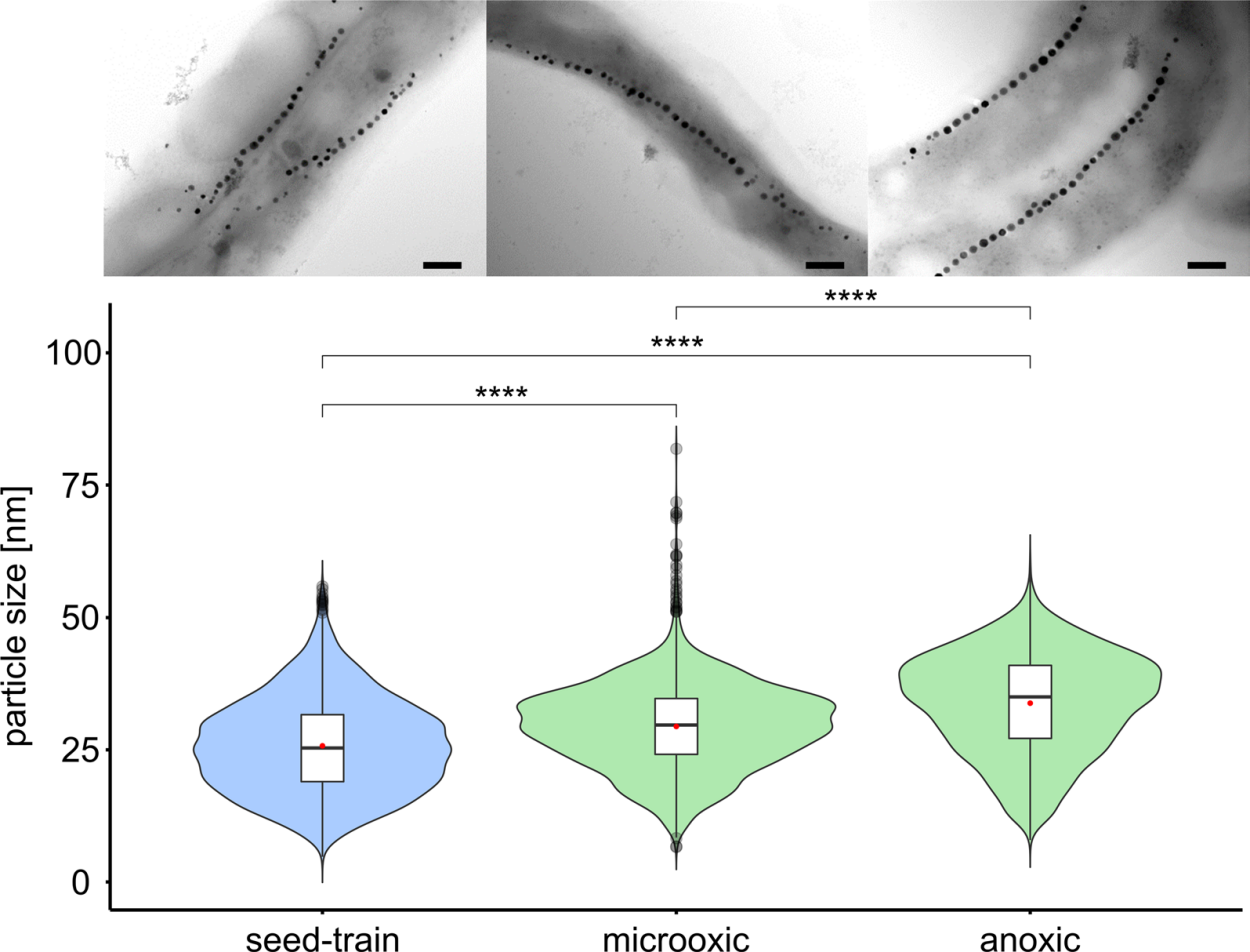
Transmission electron micrographs and magnetosome particle sizes at process end among seed-train (*n *= 2925), microoxic (1% dO_2_) (*n *= 3180) and anoxic (0% dO_2_) conditions (*n *= 3025) with representative TEM micrographs of cells under the respective conditions (scale bar 200 nm). In box plots, the box indicates the interquartile range, the bar indicates the median, and the red dot represents the mean. Grey dots represent data points above or below the 95th and 5th percentile. The violin plots show the magnetosome particle size distribution of measured particle sizes. For statistical comparison of particle sizes, Wilcoxon rank sum test was performed (****, p < 0.0001)

Taken together, if we assume, that as much as 99% of the intracellular iron content is bound in magnetite [49], the highest overall magnetite productivity of the complete process was obtained at a dO_2_ of 1% of 0.109 mg L^−1^ h^−1^ resulting from the highest maximal cell densities measured in this study (OD_565_ of 1). In contrast optimal magnetosome biosynthesis was reached anaerobically, yielding 0.048 mg L^−1^ h^−1^. Highest proportion (66.4%) of magnetosomes larger than 30 nm in diameter was obtained at 0% dO_2_, compared to 48.0% at 1% dO_2_ (Fig. 5). Furthermore, smaller magnetosomes (< 30 nm) are lost during magnetosome isolation and purification [50].

Small-angle X-ray scattering (SAXS) intensities I(q) of magnetite producing cells (0% and 1% dO_2_, Additional file 2: Figure S3 A symbols) exhibited pronounced oscillations at low and intermediate q, which are not visible in the scattering pattern of magnetosome deficient cells (95% dO_2_, Additional file 2: Figure S3 B solid lines). In contrast to these magnetosome-deficient samples, the scattering intensities of different batches of magnetosome-rich bacteria produced at identical dO_2_ conditions was remarkably reproducible concerning the size and polydispersity of the magnetosomes, which underlines the precise biological control of magnetite biomineralization (Additional file 2: Figure S3 B symbols, 3 batches, 0% dO_2_).

Although nearly no magnetosomes were detected in aerobically grown cells, differences between oxic samples (Additional file 2: Fig S3 B solid lines) in the q range of 0.01-0.15 Å^−1^ hint towards the presence of flake-like particles with a radius of about R = 10 nm and a thickness of about 3.5 nm, which may point to precursor particles of hematite resulting from the disturbed magnetite biomineralization.

## Conclusion

The streamlined seed-train and automated oxystat regime presented in this study provides well characterized and stable culturing environments for reproducible magnetosome production. By further expanding this regime with an optimized feeding strategy, future approaches can overcome growth limitations caused by substrate depletion. This would lead to higher yields with improved and reproducible magnetosome characteristics. In addition, variable oxygen control may be used to adjust the size of magnetite particles with distinct magnetic characteristics ‘tailored’ for the desired application. The future development of high-yield fermentation protocols combined with high process reproducibility and magnetosome characteristics, will pave the way for industrial production for wide-spread application in various fields.

## Materials and methods

### Bacterial strains

*Magnetospirillum gryphiswaldense* strain MSR-1 (DSM 6361; [28, 51]) was used in all experiments.

### Determination of storage influence on cell viability

For investigation of effects on cell viability after prolonged storage at 4 °C, the number of passages, which are needed for minimal lag-phase length, was determined. Therefore, cells were inoculated from 4 °C stock cultures in 30 mL FSM in 50 mL conical centrifuge tubes incubated shaking at 120 rpm and 28 °C for 24 h. After each passage (maximal 4 passages), cell growth was monitored with an initial OD_565_ of 0.01 using an Infinite 200pro microplate reader (Tecan, Männedorf, Switzerland) in 24-well microtiter plates with 1 mL FSM and incubated shaking at 28 °C. In case of first passage, growth experiments were inoculated directly from 4 °C stock cultures.

### Seed-train standardization

We first sought to establish a robust seed-train under microoxic conditions by optimizing scale up, incubation conditions, aeration (by variation of agitation, headspace to volume ratio, and closed lids vs. free exchange with air) and media composition (peptone and iron concentrations). Microoxic conditions were chosen for practical reasons, since they are known to provide a reasonable trade-off between growth and magnetosome biomineralization, whereas anoxic and oxic conditions cause limited or impaired growth, respectively [32].

As first step of the seed-train, *M. gryphiswaldense* cells were incubated at 24 °C in 15 mL conical centrifuge tubes with 10 mL flask standard medium (FSM) comprising: 10 mM 2-[4-(2-hydroxyethyl)piperazin-1-yl]ethanesulfonic acid (HEPES) (pH 7.0), 15 mM potassium lactate, 4 mM NaNO_3_, 0.74 mM KH_2_PO_4_, 0.6 mM MgSO_4_ x 7H_2_O, 50 µM iron citrate, 3 g L^−1^ soy peptone and 0.1 g L^−1^ yeast extract [32] . For cultivation in screw-cap bottles, preculture medium was used comprising of 10 mM HEPES (pH 7.0), 15 mM potassium lactate, 4 mM NaNO_3_, 0.74 mM KH_2_PO_4_, 0.6 mM MgSO_4_ × 7H_2_O, 150 µM iron citrate, 1 g L^−1^ soy peptone and 0.1 g L^−1^ yeast extract.

For standardization of the flask cultivation, growth and *C*_mag_ were tested of cultures cultivated in 50 mL and 100 mL firmly closed screw-capped bottles with 30 mL medium incubated at 24 °C for 24, 40 and 48 h. Cultures were inoculated 1:10 with cells cultured in 15 mL tubes as described before. Samples were taken at the end of incubation to not disturb growth conditions by the sampling procedure. The same experimental setup was used for cultivation in 500 mL and 1000 mL slightly opened screw-capped bottles with 300 mL medium incubated at 28 °C for 16 or 20 h.

For routine cultivation, liquid stock cultures of 10 mL are usually stored at 4 °C, which however gradually decreases viability after prolonged storage (4–9 weeks) as observed in growth experiments (Additional file 3: Figure S1). We found that incubation of two successive passages at 28 °C for 24 h with slight agitation exposed to air resulted in efficient reactivation and robust outgrowth. Accordingly, the two passages were adapted for initial subcultures incubated in 10 mL at room temperature (24 °C) for 40 h, followed by one step in a 100 mL screw-capped bottle with 30 mL medium at 24 °C for 40 h and one step in a 500 mL slightly opened screw-capped bottle with 300 mL at 28 °C for 16 h. All culturing steps described in this section were inoculated with a culture to medium ratio of 1/10 and incubated at 120 rpm in an orbital shaking incubator.

### Oxystat cultivation

A stirred-tank 3 L jacketed bioreactor was employed in this study (Bioflo™ 320, Eppendorf Bioprocess, Jülich, Germany) equipped with four baffles and a stirrer with one pitched-blade impeller at the end of the agitator shaft and one Rushton impeller 4 cm above the pitched-blade impeller. Two cable ties were installed at the headspace part of the stirrer shaft leading to adequate, mechanical foam dispersion in fermentation experiments. The control units of the fermenter system were equipped with thermal mass flow controllers for gas inlet.

During the process, pH was monitored online with an InPro3253i (Mettler-Toledo, Columbus, USA) pH probe and was controlled at pH = 7 ± 0.1 by automated addition of 1 M H_2_SO_4_ to compensate for the basification during main growth or 1 M KOH in stationary phase. Oxygen concentration was measured online with an InPro6850i (Mettler-Toledo, Columbus, USA) O_2_ sensor with a lower limit of 6 parts per billion (ppb) an accuracy of ± 1%. After sterilization and cooling of the fermenter vessel to process temperature (28 °C), the medium was sparged first with nitrogen (dO_2_ 0%) until measured raw current values were stable prior to zero calibration, followed by sparging with air to saturation (dO_2_ 100%) for O_2_ probe calibration.

Cultivation in bioreactors was carried out in 2.8 L of large-scale medium (LSM) comprising 15 mM potassium lactate, 4 mM NaNO_3_, 0.74 mM KH_2_PO_4_, 0.6 mM MgSO_4_ x 7H_2_O, 150 µM iron citrate, 3 g L^−1^ soy peptone and 0.1 g L^−1^ yeast extract. For anaerobic fermentation processes, additional sodium nitrate was supplemented to 10 mM to prolong the main growth phase due to the increased nitrate requirement. Prior to inoculation of anoxic and microoxic processes, oxygen was gassed out with nitrogen. During anaerobic processes, the medium was continuously sparged with 0.2 SLPM nitrogen to prevent oxygen diffusion into the system. Agitation was kept constant at 100 rpm. For processes under controlled oxygen conditions (dO_2_ set point 1% and 95%), a programmed cascade controlled dO_2_ by automated adjustment of agitation (100–300 rpm) and airflow (0–10 SLPM) with compressed air (see results for cascade specifications).

### Cell growth and magnetic response

Cell growth and magnetic response was monitored turbidimetrically by measuring the optical density at 565 nm (OD_565_) with an Ultrospec2000 pro spectrophotometer. Magnetic response of the culture was measured as described in Schüler et al. [29]. Briefly, cells were magnetically aligned perpendicular and vertical to the light beam of a photometer resulting in a change of the OD_565_. The ratio of maximal and minimal scattering intensities subtracted by 1 (*C*_mag_) represents the magnetic response of the cells as estimation for magnetosome biomineralization.

### Substrate monitoring

The lactate concentration was measured with the handheld device DiaSpect Tm (EKF Diagnostics, Germany) according manufacturer’s instructions.

The nitrate concentration was measured using the Szechrome NAS reagent (Polysciences inc., Warrington, USA) according to manufacturer’s instructions. Briefly, the working solution was prepared by mixing 85–86% phosphoric acid and 95–97% sulfuric acid in equimolar amounts. Afterwards 5 g L^−1^ reagent were added and mixed until the reagent was completely dissolved. Samples were diluted with ddH_2_O to the expected sensitivity ratio (1–20 mg L^−1^) of the reagent, followed by mixing of 100 µL of diluted sample with 1000 µL of the working solution in a cuvette. After 5 min of incubation, the absorption at 570 nm was measured using an Ultrospec2000 pro spectrophotometer.

Nitrite concentration was determined by using the Griess reagent kit (Sigma-Aldrich, st. Louis, USA) according to manufacturer’s instructions. Briefly, 1 g of Griess reagent was mixed with 25 mL ddH_2_O. Afterwards, 500 µL of the working solution were mixed with 500 µL of the sample and after 5 min of incubation, measured photometrically at 540 nm.

### Determination of iron content

To follow iron enrichment within cell pellets of *M. gryphiswaldense*, iron content was determined by atomic absorption spectroscopy (AAS) throughout the cultivation. 10 mL of fermentation broth from the bioreactor were pelleted at 3700 g for 10 min at room temperature using an Allegra^®^ X-15R centrifuge (Beckman Coulter, Brea, USA). The pellet was resuspended in 5 mL 0.5 M HEPES pH 7.0 and was subsequently analyzed using an Analytic Jena contrAA300 high-resolution atomic absorption spectrometer (Analytik Jena, Jena, Germany) equipped with a 300 W xenon short-arc lamp (XBO 301, GLE, Berlin, Germany) as continuum radiation source. Detection was carried out with a compact high-resolution double monochromator and a charge-coupled device (CCD) array detector with a resolution of 2 pm per pixel in the far ultraviolet range. The wavelength for detection was set to 248.3 nm using an oxidizing air/acetylene flame. The measured values are given in mean values representing averaged values from three experiments measured in technical quintuplicates.

### Transmission electron microscopy (TEM)

Transmission electron microscopy of whole cells was performed with specimens directly deposited onto carbon-coated copper grids (Science Services, Munich, Germany). For TEM imaging a Jeol Jem 1400 + (Freising, Germany) was operated at an acceleration voltage of 80 kV. Image acquisition was performed with a Gatan Erlangshen ES500W CCD camera. Average particle sizes were measured by data processing with ImageJ software package v1.52i. For quantitative assessment of magnetite particle biomineralization at different timepoints, specifically at the process start, in the mid-exponential growth phase and at end of growth 1000 magnetosome particles per triplicate were counted and combined for evaluation.

### Small-angle X-ray scattering (SAXS)

Nanostructural investigation of magnetosomes was performed as described in Rosenfeldt et al. [43]. Briefly, harvested cells were centrifuged at 8300 g for 10 min using a Sorvall RC-5B Plus centrifuge (Thermo Fisher Scientific, Waltham, USA), resuspended in 50 mM HEPES buffer pH 7.0 and filled into glass capillaries (ø = 1 mm, Hilgenberg, Germany). Samples were measured using a Double Ganesha AIR system (SAXSLAB, Skovlunder, Denmark). A rotating anode (Cu, MicroMax 007HF, Rigaku Corporation, Japan) served as source for monochromatic radiation with a wavelength of λ = 1.54 Å. Two dimensional scattering patterns were recorded with a position-sensitive detector (PILATUS 300 K, Dectris) and converted into 1-dimensional intensity profiles of I(q) vs q by radial averaging. The obtained 1D-SAXS data were normalized to accumulation time, sample thickness and transmission before subtraction of the scattering contributions of the solvent. A glass capillary filled with HEPES buffer was used for background correction. The scattering curves were analyzed based on a model of monodispersed, non-interacting spheres arranged in a chain using the software SasView 4.2.

### Statistical analyses

Group data are reported as mean ± standard deviation. For determination of statistical significance, Wilcoxon rank sum test was performed using R-software.

## Supplementary information

**Additional file 1: Figure S1.** Influence of storage duration in weeks on cell viability.

**Additional file 2: Figure S2.** Oxystat fermentations at 1% dO_2_.

**Additional file 3: Figure S3.** Representative small-angle X-ray scattering curves of *M. gryphiswaldense* cells.

## Data Availability

Not applicable.

## References

[1] Lefèvre CT, Bazylinski DA (2013). Ecology, diversity, and evolution of magnetotactic bacteria. Microbiol Mol Biol Rev.

[2] Uebe R, Schüler D (2016). Magnetosome biogenesis in magnetotactic bacteria. Nat Rev Microbiol.

[3] Barber-Zucker S, Keren-Khadmy N, Zarivach R (2015). From invagination to navigation: the story of magnetosome-associated proteins in magnetotactic bacteria. Protein Sci.

[4] Lohße A, Ullrich S, Katzmann E, Borg S, Wanner G, Richter M, Voigt B, Schweder T, Schüler D (2011). Functional analysis of the magnetosome island in *Magnetospirillum gryphiswaldense*: the *mamAB* operon is sufficient for magnetite biomineralization. PLoS ONE.

[5] Lohße A, Borg S, Raschdorf O, Kolinko I, Tompa É, Pósfai M, Faivre D, Baumgartner J, Schüler D (2014). Genetic dissection of the *mamAB* and *mms6* operons reveals a gene set essential for magnetosome biogenesis in *Magnetospirillum gryphiswaldense*. J Bacteriol.

[6] Staniland SS, Ward B, Harrison A, van der Laan G, Telling N (2007). Rapid magnetosome formation schown by real-time X-ray magnetic circular dichroism. Proc Natl Acad Sci USA..

[7] Staniland SS, Rawlings AE (2016). Crystallizing the function of the magnetosome membrane mineralization protein Mms6. Biochem Soc Trans.

[8] Hergt R, Hiergeist R, Zeisberger M, Schüler D, Heyen U, Hilger I, Kaiser WA (2005). Magnetic properties of bacterial magnetosomes as diagnostic and therapeutic tools. J Magn Magn Matter..

[9] Alphandéry E, Guyot F, Chebbi I (2012). Preparation of chains of magnetosomes, isolated from *Magnetospirillum magneticum* strain AMB-1 magnetotactic bacteria, yielding efficient treatment of tumors using magnetic hyperthermia. Int J Pharm.

[10] Gandia D, Gandarias L, Rodrigo I, Robles-García, Das R, Garaio E, García JA, Phan MH, Srikanth H, Orue I, Alonso J, Muela A, Fdez-Gubieda (2019). Unlocking the potential of magnetotactic bacteria as magnetic hyperthermia agents. Small.

[11] Sangnier AP, Preveral S, Curcio A, Silva AKA, Lefèvre C, Pignol D, Lalatonne Y, Wilhelm C (2018). Targeted thermal therapy with genetically engineered magnetic magnetosomes@RGD: photothermia is far more efficient than magnetic hyperthermia. J Control Release.

[12] Hafsi M, Preveral S, Hoog C, Hérault J, Perrier GA, Lefèvre CT, Michel H, Pignol D, Doyen J, Pourcher T, Humbert O, Thariat J, Cambien B (2020). RGD-functionalized magnetosomes are efficient tumor radioenhancers for X-rays and protons. Nanomedicine NBM.

[13] Mériaux S, Boucher M, Marty B, Lalatonne Y, Prévéral S, Motte L, Lefèvre CT, Geffroy, Lethimonnier F, Péan M, Garcia D, Adryanczyk-Perrier G, Pignol D, Ginet N (2015). Magnetosomes, biogenic magnetic nanomaterials for brain molecular imaging with 17.2 T MRI scanner. Adv Healthc Mater..

[14] Orlando T, Mannucci S, Fantechi E, Conti G, Tambalo S, Busato A, Innocenti C, Ghin L, Bassi R, Arosio P, Orsini F, Sangregorio C, Corti M, Casula MF, Marzola P, Lascialfari A, Sbarbati A (2016). Characterization of magnetic nanoparticles from *Magnetospirillum gryphiswaldense* as potential theranostics tools. Contrast Media Mol Imaging.

[15] Heinke D, Kraupner A, Eberbeck D, Schmidt D, Radon P, Uebe R, Schüler D, Briel A (2017). MPS and MRI efficacy of magnetosomes from wild-type and mutant bacterial strains. Int J Magn Part Imaging..

[16] Kraupner A, Eberbeck D, Heinke D, Uebe R, Schüler D, Briel A (2017). Bacterial magnetosomes–nature’s powerful contribution to MPI tracer research. Nanoscale..

[17] Tanaka T, Takeda H, Ueki F, Obata K, Tajima H, Takeyama H, Goda Y, Fujimoto S, Matsunaga T (2004). Rapid and sensitive detection of 17β-estradiol in environmental water using automated immunoassay system with bacterial magnetic particles. J Biotechnol.

[18] Matsunaga T, Kamiya S (1987). Use of magnetic particles isolated from magnetotactic bacteria for enzyme immobilization. Appl Microbiol Biotechnol.

[19] Ginet N, Pardoux R, Adryanczyk G, Garcia D, Brutesco C, Pignol D (2011). Single-step production of a recyclable nanobiocatalyst for organophosphate pesticides biodegradation using functionalized bacterial magnetosomes. PLoS ONE.

[20] Mickoleit F, Lanzloth C, Schüler D (2020). A versatile toolkit for controllable and highly selective multifunctionalization of bacterial magnetic nanoparticles. Small.

[21] Honda T, Yasuda T, Tanaka T, Hagiwara K, Arai T, Yoshino T (2014). Functional expression of full-length TrkA in prokaryotic host *Magnetospirillum magneticum* AMB-1 by using a magnetosome display system. Appl Environ Microbiol.

[22] Xu J, Hu J, Liu L, Li L, Wang X, Zhang H, Jiang W, Tian J, Li Y, Li J (2014). Surface expression of protein A on magnetosomes and capture of pathogenic bacteria by magnetosome/antibody complexes. Front Microbiol..

[23] Xu J, Liu L, He J, Ma S, Li S, Wang Z, Xu T, Jiang W, Wen Y, Li Y, Tian J, Li FJ (2019). Engineered magnetosomes fused to functional molecule (protein A) provide a highly effective alternative to commercial immunomagnetic beads. Nanobiotechnol..

[24] Wacker R, Ceyhan B, Alhorn P, Schüler D, Lang C, Niemeyer CM (2003). Magneto Immuno-PCR: a novel immunoassay based on biogenic magnetosome nanoparticles. Biochem Biophy Res Commun..

[25] Mickoleit F, Jérôme V, Freitag R, Schüler D (2020). Bacterial magnetosomes as novel platform for the presentation of immunostimulatory, membrane-bound ligands in cellular biotechnology. Adv Biosyst..

[26] Borg S, Rothenstein D, Bill J, Schüler D (2015). Generation of multi-shell magnetic hybrid nanoparticles by encapsulation of genetically engineered and fluorescent bacterial magnetosomes with ZnO and SiO2. Small.

[27] Mickoleit F, Borkner CB, Toro-Nahuelpan M, Herold HM, Maier DS, Plitzko JM, Scheibel T, Schüler D (2018). In vivo coating of bacterial magnetic nanoparticles by magnetosome expression of spider silk-inspired peptides. Biomacromol.

[28] Schleifer KH, Schüler D, Spring S, Weizenegger M, Amann R, Ludwig W, Köhler M (1991). The genus *Magnetospirillum* gen. nov. description of *Magnetospirillum gryphiswaldense* sp. nov. and transfer of *Aquaspirillum magnetotacticum* to *Magnetospirillum magnteotacticum* comb. nov. Syst Appl Microbiol..

[29] Schüler D, Uhl R, Bäuerlein E (1995). A simple light scattering method to assay magnetism in *Magnetospirillum gryphiswaldense*. FEMS Microbiol Lett.

[30] Schüler D, Bäuerlein E (1998). Dynamics of iron uptake and Fe_3_O_4_ biomineralization during aerobic and microaerobic growth of *Magnetospirillum gryphiswaldense*. J Bacteriol.

[31] Katzmann E, Eibauer M, Lin W, Pan Y, Plitzko JM, Schüler D (2013). Analysis of magnetosome chains in magnetotactic bacteria by magnetic measurements and automated image analysis of electron micrographs. Appl Environ Microbiol.

[32] Heyen U, Schüler D (2003). Growth and magnetosome formation by microaerophilic *Magnetospirillum* strains in an oxygen-controlled fermentor. Appl Microbiol Biotechnol.

[33] Li Y, Katzmann E, Borg S, Schüler D (2012). The periplasmic nitrate reductase Nap is required for anaerobic growth and involved in redox control of magnetite biomineralization in *Magnetospirillum gryphiswaldense*. J Bacteriol.

[34] Li Y, Sabaty M, Borg S, Silva KT, Pignol D, Schüler D (2014). The oxygen sensor MgFnr controls magnetite biomineralization by regulation of denitrification in *Magnetospirillum gryphiswaldense*. BMC Microbiol.

[35] Li Y, Raschdorf O, Silva KT, Schüler D (2014). The terminal oxidase cbb3 functions in redox control of magnetite biomineralization in *Magnetospirillum gryphiswaldense*. J Bacteriol.

[36] Sun JB, Zhao F, Tang T, Jiang W, Tian JS, Li Y, Li JL (2008). High-yield growth and magnetosome formation by *Magnetospirillum gryphiswaldense* MSR-1 in an oxygen-controlled fermentor supplied solely with air. Appl Microbiol Biotechnol.

[37] Liu Y, Li GR, Guo FF, Jiang W, Li Y, Li LJ (2010). Large-scale production of magnetosomes by chemostat culture of *Magnetospirillum gryphiswaldense* at high cell density. Microb Cell Fact.

[38] Zhang Y, Zhang X, Jiang W, Li Y, Li J (2011). Semicontinuous culture of *Magnetospirillum gryphiswaldense* MSR-1 cells in an autofermentor by nutrient-balanced and isosmotic feeding strategy. Appl Environ Microbiol.

[39] Fernández-Castané A, Li H, Thomas ORT, Overton TW (2018). Development of a simple intensified fermentation strategy for growth of *Magnetospirillum gryphiswaldense* MSR-1: physiological responses to changing environmental conditions. New Biotechnol.

[40] Fernández-Castané A, Li H, Thomas OR, Overton TW (2017). Flow cytometry as a rapid analytical tool to determine physiological responses to changing O_2_ and iron concentration by *Magnetospirillum gryphiswaldense* strain MSR-1. Sci Rep..

[41] Berny C, Le Fèvre R, Guyot F, Blondeau K, Guizonne C, Rousseau E, Bayan N, Alphandéry E (2020). A method for producing highly pure magnetosomes in large quantity for medical applications using *Magnetospirillum gryphiswaldense* MSR-1 magnetotactic bacteria amplified in minimal growth media. Front Bioeng Biotechnol..

[42] Zhuang S, Anyaogu DC, Kasama T, Workman M, Mortensen UH, Hobley TJ (2017). Effects of dissolved oxygen concentration and iron addition on immediate-early gene expression of *Magnetospirillum gryphiswaldense* MSR-1. FEMS Microbiol Lett..

[43] Rosenfeldt S, Riese CN, Mickoleit F, Schüler D, Schenk AS (2019). Probing the nanostructure of bacterial magnetosomes by small-angle X-ray scattering. Appl Environ Microbiol.

[44] Gogate PR, Beenackers AACM, Pandit AB (2000). Multiple-impeller systems with a special emphasis on bioreactors: a critical review. Biochem Eng J.

[45] Reitzer L (2003). Nitrogen assimilation and global regulation in *Escherichia coli*. Annu Rev Microbiol.

[46] Chubukov V, Gerosa L, Kochanowski K, Sauer U (2014). Coordination of microbial metabolism. Nat Rev Microbiol.

[47] Blakemore RP, Short KA, Bazylinski DA, Rosenblatt C, Frankel RB (1985). Microaerobic conditions are required for magnetite formation within *Aquasprillum magnetotacticum*. Geomicrobiol J.

[48] Abdelrazig S, Safo L, Rance GA, Fay MW, Theodosiou E, Topham PD, Kim DH, Fernández-Castané A (2020). Metabolic characterisation of *Magnetospirillum gryphiswaldense* MSR-1 using LC-MS-based metabolite profiling. RSC Adv..

[49] Grünberg K, Müller EC, Otto A, Reszka R, Linder D, Kube M, Reinhardt R, Schüler D (2004). Biochemical and proteomic analysis of the magnetosome membrane in *Magnetospirillum gryphiswaldense*. Appl Environ Microbiol.

[50] Rosenfeldt S, Mickoleit F, Jörke C, Clement JH, Markert S, Jérôme V, Schwarzinger S, Freitag R, Schüler D, Uebe R, Schenk AS (2020). Towards standardized purification of bacterial magnetic nanoparticles for future in vivo applications. Acta Biomater.

[51] Schüler D, Köhler M (1992). The isolation of a new magnetic spirillum. Zentralbl Mikrobiol..

