# Supplementary figures and images for: An automated oxystat fermentation regime for microoxic cultivation of Magnetospirillum gryphiswaldense
Source: Microb Cell Fact. 2020 Nov 10;19:206. doi: 10.1186/s12934-020-01469-z (PMC7654035; doi:10.1186/s12934-020-01469-z)

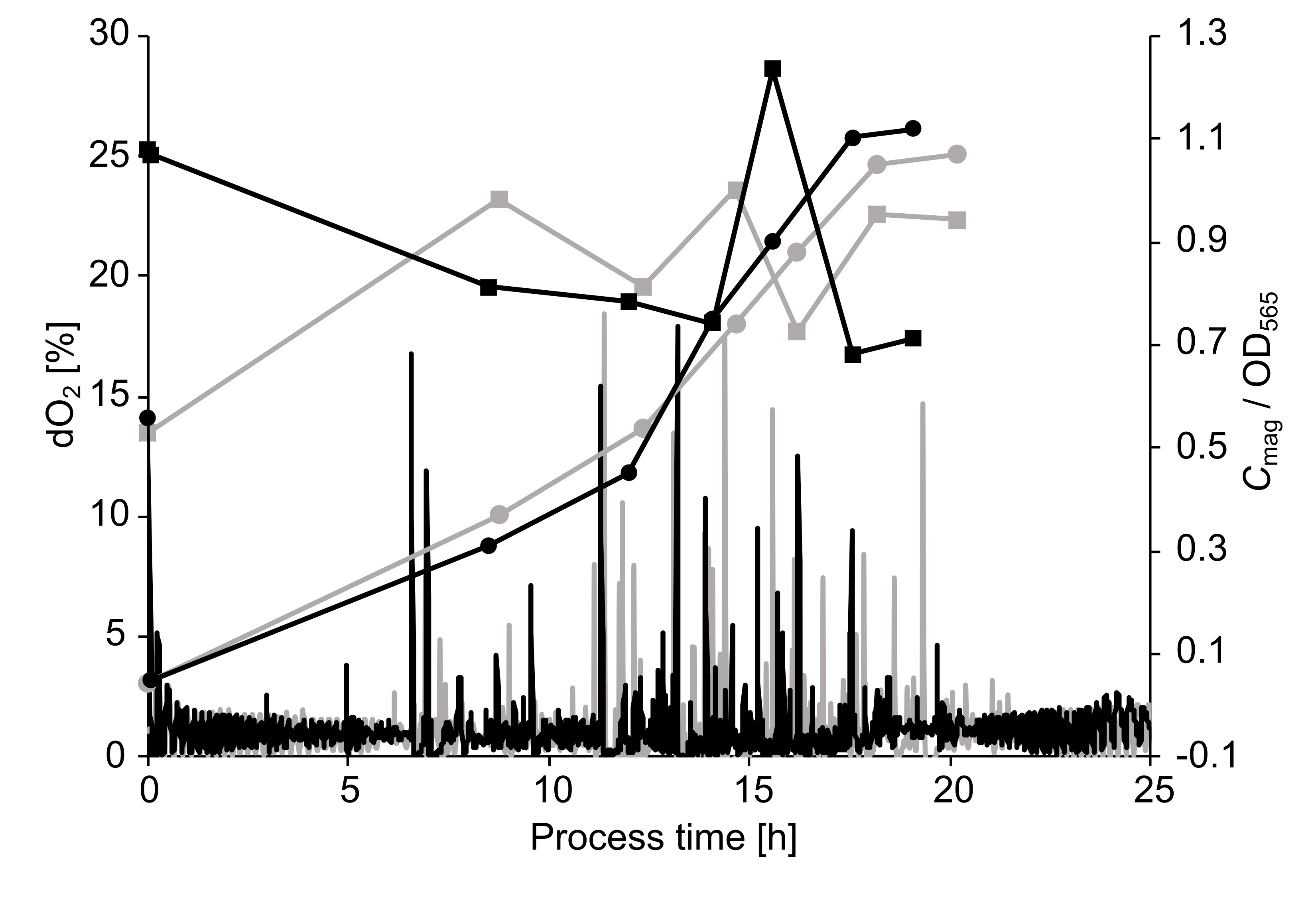

Supplement: Supplementary file 1 — Additional file 1: Figure S1. Influence of storage duration in weeks on cell viability. [file 12934_2020_1469_MOESM1_ESM.png]

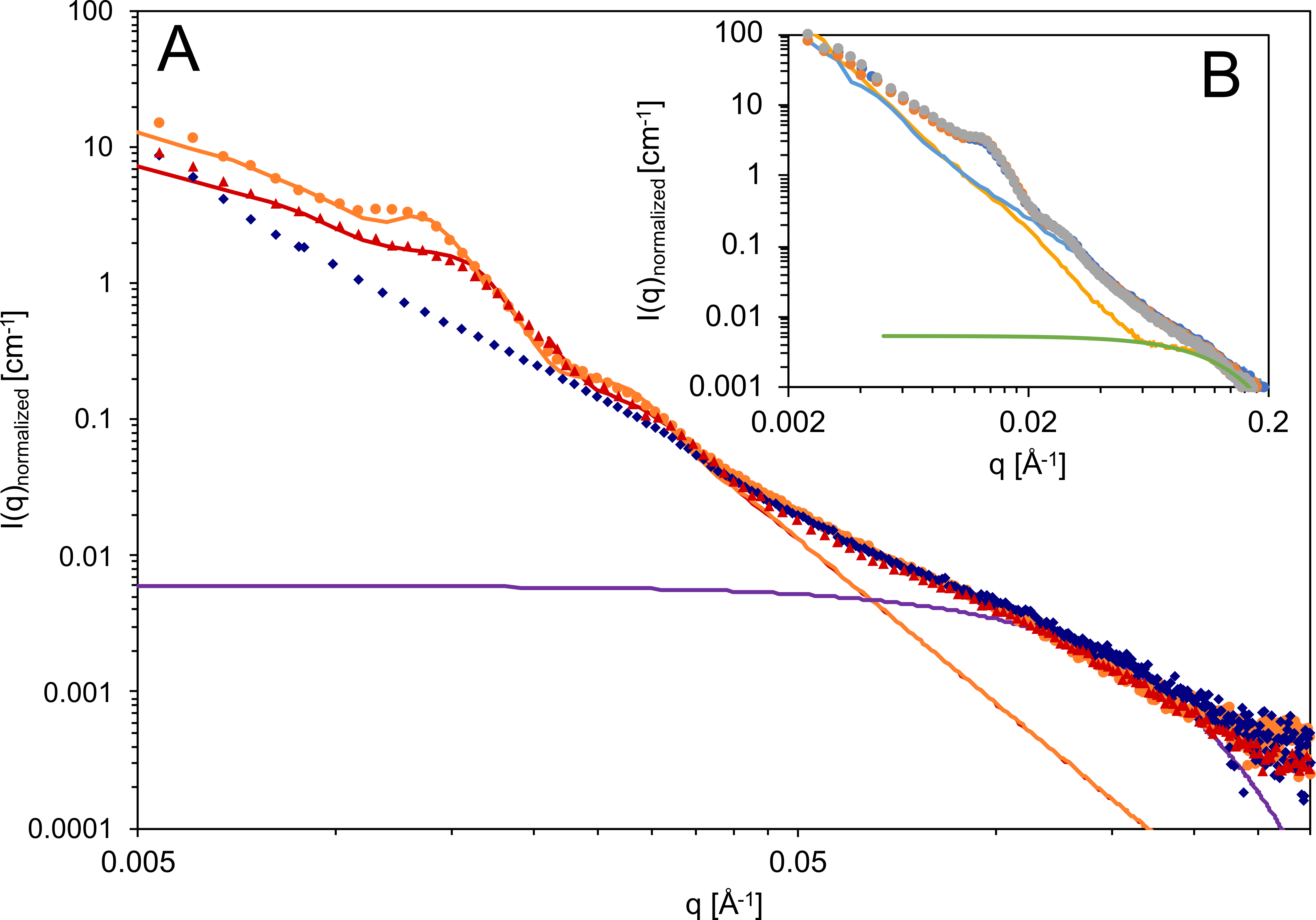

Supplement: Supplementary file 2 — Additional file 2: Figure S2. Oxystat fermentations at 1% dO2. [file 12934_2020_1469_MOESM2_ESM.png]

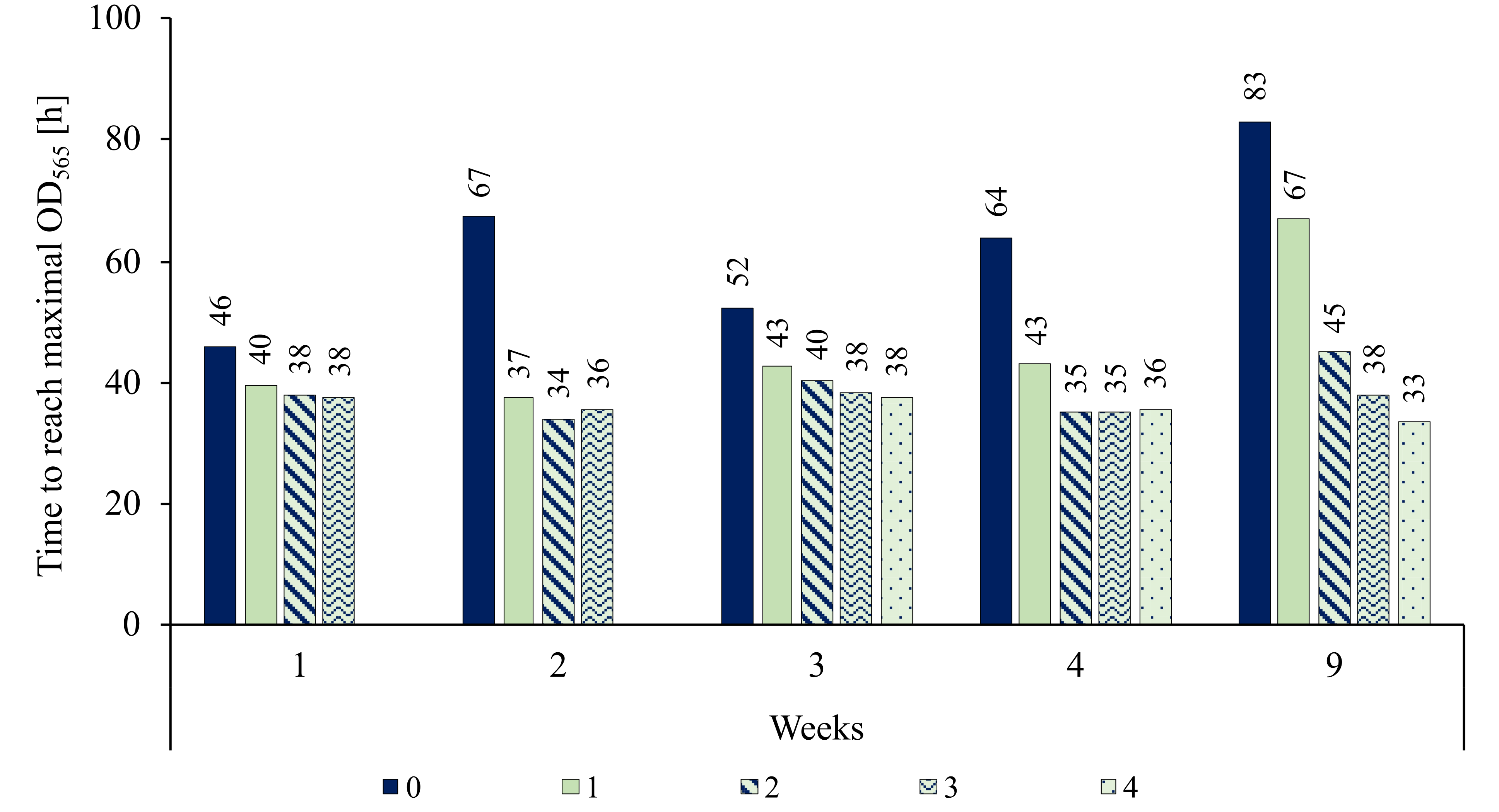

Supplement: Supplementary file 3 — Additional file 3: Figure S3. Representative small-angle X-ray scattering curves of M. gryphiswaldense cells. [file 12934_2020_1469_MOESM3_ESM.png]
